# Detailed statistical analysis plan for a secondary Bayesian analysis of the SafeBoosC-III trial: a multinational, randomised clinical trial assessing treatment guided by cerebral oximetry monitoring versus usual care in extremely preterm infants

**DOI:** 10.1186/s13063-023-07720-3

**Published:** 2023-11-16

**Authors:** Markus Harboe Olsen, Mathias Lühr Hansen, Theis Lange, Christian Gluud, Lehana Thabane, Gorm Greisen, Janus Christian Jakobsen, the SafeBoosC-III Trial Group, Adelina Pellicer, Afif El-Kuffash, Agata Bargiel, Ana Alarcon, Andrew Hopper, Anita Truttmann, Anja Hergenhan, Anja Klamer, Anna Curley, Anne Marie, Anne Smits, Asli Cinar Memisoglu, Barbara Krolak-Olejnik, Beata Rzepecka, Begona Loureiro Gonzales, Beril Yasa, Berndt Urlesberger, Catalina Morales-Betancourt, Chantal Lecart, Claudia Knöepfli, Cornelia Hagmann, David Healy, Ebru Ergenekon, Eleftheria Hatzidaki, Elena Bergon-Sendin, Eleni Skylogianni, Elzbieta Rafinska-Wazny, Emmanuele Mastretta, Eugene Dempsey, Eva Valverde, Evangelina Papathoma, Fabio Mosca, Gabriel Dimitriou, Gerhard Pichler, Giovanni Vento, Gitte Holst Hahn, Gunnar Naulaers, Guoqiang Cheng, Hans Fuchs, Hilal Ozkan, Isabel De Las Cuevas, Itziar Serrano-Vinuales, Iwona Sadowska-Krawczenko, Jachym Kucera, Jakub Tkaczyk, Jan Miletin, Jan Sirc, Jana Baumgartner, Jonathan Mintzer, Julie De Buyst, Karen McCall, Konstantina Tsoni, Kosmas Sarafidis, Lars Bender, Laura Serrano Lopez, Le Wang, Liesbeth Thewissen, Lin Huijia, Lina Chalak, Ling Yang, Luc Cornette, Luis Arruza, Maria Wilinska, Mariana Baserga, Marie Isabel Skov Rasmussen, Marta Mencia Ybarra, Marta Teresa Palacio, Martin Stocker, Massimo Agosti, Merih Cetinkaya, Miguel Alsina, Monica Fumagalli, Munaf M. Kadri, Mustafa Senol Akin, Münevver Baş, Nilgun Koksal, Olalla Otero Vaccarello, Olivier Baud, Pamela Zafra, Peter Agergaard, Peter Korcek, Pierre Maton, Rebeca Sanchez-Salmador, Ruth del Rio Florentino, Ryszard Lauterbach, Salvador Piris Borregas, Saudamini Nesargi, Serife Suna, Shashidhar Appaji Rao, Shujuan Zeng, Silvia Pisoni, Simon Hyttel-Sørensen, Sinem Gulcan Kersin, Siv Fredly, Suna Oguz, Sylwia Marciniak, Tanja Karen, Tomasz Szczapa, Tone Nordvik, Veronika Karadyova, Xiaoyan Gao, Xin Xu, Zachary Vesoulis, Zhang Peng, Zhaoqing Yin

**Affiliations:** 1 Centre for Clinical Intervention Research, Copenhagen Trial Unit, The Capital Region, Copenhagen University Hospital ─ Rigshospitalet, Copenhagen, Denmark; 2 Department of Neuroanaesthesiology, Neuroscience Centre, Copenhagen University Hospital ─ Rigshospitalet, Copenhagen, Denmark; 3 Department of Neonatology, Juliane Marie Centre, Copenhagen University Hospital ─ Rigshospitalet, Copenhagen, Denmark; 4 Section of Biostatistics, Department of Publich Health, Copenhagen University, Øster Farimagsgade 5, Copenhagen K, Denmark; 5 The Faculty of Health Sciences, Institute of Regional Health Research, University of Southern Denmark, Odense, Denmark; 6 Health Research Methods, Evidence, and Impact, McMaster University, Hamilton, ON Canada; 7 Biostatistics Unit, St Joseph’s Healthcare—Hamilton, Hamilton, ON Canada; 8 Faculty of Health Sciences, University of Johannesburg, Johannesburg, South Africa

**Keywords:** Randomised clinical trial, Extremely preterm, Cerebral oximetry, Near-infrared spectroscopy, Statistical analysis plan, Bayesian statistics

## Abstract

**Background:**

Extremely preterm infants have a high mortality and morbidity. Here, we present a statistical analysis plan for secondary Bayesian analyses of the pragmatic, sufficiently powered multinational, trial—SafeBoosC III—evaluating the benefits and harms of cerebral oximetry monitoring plus a treatment guideline versus usual care for such infants.

**Methods:**

The SafeBoosC-III trial is an investigator-initiated, open-label, randomised, multinational, pragmatic, phase III clinical trial with a parallel-group design. The trial randomised 1601 infants, and the frequentist analyses were published in April 2023. The primary outcome is a dichotomous composite outcome of death or severe brain injury. The exploratory outcomes are major neonatal morbidities associated with neurodevelopmental impairment later in life: (1) bronchopulmonary dysplasia; (2) retinopathy of prematurity; (3) late-onset sepsis; (4) necrotising enterocolitis; and (5) number of major neonatal morbidities (count of bronchopulmonary dysplasia, retinopathy of prematurity, and severe brain injury). The primary Bayesian analyses will use non-informed priors including all plausible effects. The models will use a Hamiltonian Monte Carlo sampler with 1 chain, a sampling of 10,000, and at least 25,000 iterations for the burn-in period. In Bayesian statistics, such analyses are referred to as ‘posteriors’ and will be presented as point estimates with 95% credibility intervals (CrIs), encompassing the most probable results based on the data, model, and priors selected. The results will be presented as probability of any benefit or any harm, Bayes factor, and the probability of clinical important benefit or harm. Two statisticians will analyse the blinded data independently following this protocol.

**Discussion:**

This statistical analysis plan presents a secondary Bayesian analysis of the SafeBoosC-III trial. The analysis and the final manuscript will be carried out and written after we publicise the primary frequentist trial report. Thus, we can interpret the findings from both the frequentists and Bayesian perspective. This approach should provide a better foundation for interpreting of our findings.

**Trial registration:**

ClinicalTrials.org, NCT03770741. Registered on 10 December 2018.

**Supplementary Information:**

The online version contains supplementary material available at 10.1186/s13063-023-07720-3.

## Introduction

Extremely preterm infants have a high mortality and morbidity [1, 2]. The SafeBoosC-II trial observed that cerebral oximetry monitoring by near-infrared spectroscopy (NIRS), plus a treatment guideline for the first three days of life, could potentially reduce the cerebral hypoxic burden [3]. There were also a trend towards reduced mortality and occurrence of severe brain injury in the intervention group, whereas the occurrence of bronchopulmonary dysplasia and retinopathy of prematurity tended to increase in this group [4]. As the SafeBoosC-II trial was insufficiently powered to detect a difference on these clinical outcomes, a larger trial was needed. Therefore, the pragmatic, multinational trial—the SafeBoosC III trial—evaluating the benefits and harms of cerebral oximetry monitoring plus an accompanying treatment guideline versus usual care—was conducted [5, 6].

The primary analyses of the SafeBoosC-III trial were carried out using frequentist statistical methods [7]. Bayesian statistical analyses are now more commonly used both as a standalone analysis of randomised clinical trials, primarily those of adaptive design but also as a sensitivity analysis of sequentially randomised clinical trials [8–11]. Previously, Bayesian analyses have nuanced the conventional frequentists statistics interpretation when the *p* values have been used with a dichotomous threshold of difference and interpreted to prove or disprove similarity between groups [12, 13].

Here, we present a secondary statistical analysis plan for a sensitivity analysis with a pre-defined statistical code of the SafeBoosC-III trial using Bayesian statistical analyses.

## Methods

The SafeBoosC-III trial is an investigator-initiated, open-label, randomised, multinational, pragmatic, phase-III superiority clinical trial with a parallel-group design [5]. The trial methodology and design has previously been described in detail elsewhere [5]. The trial aims to evaluate the benefits and harms of cerebral oximetry monitoring by spatially resolved near-infrared spectroscopy (NIRS) plus a treatment guideline versus usual care. A total of 1601 infants were randomised with an allocation ratio of 1:1, stratified by site and gestational age (< 26 weeks compared to ≥ 26 weeks). Randomisation and initiation of cerebral oximetry monitoring should occur within 6 h of birth, and cerebral oximetry monitoring should continue for the first 72 h after birth [5]. The trial was registered on ClinicalTrials.gov (identification no. NCT03770741) before inclusion of the first participant on 10 December 2018. The consent workflow and eligibility criteria are described elsewhere [5]. Overall, all infants born before 28 weeks postmenstrual age with decision to provide full life support and with the possibility to initiate cerebral oximetry monitoring within 6 h after birth were eligible [5].

### Trial status

The last participant was included 16 December 2021, and the primary frequentists analyses were carried out on 30 May 2022, and the main article has been published [6].

### Outcomes

All outcomes are assessed when the infant is discharged from the hospital, at 36 weeks of postmenstrual age, or when the infant dies, whichever event occurs first. The primary outcome is a dichotomous composite outcome of death or severe brain injury, defined as one of the following: cerebral haemorrhage grade ≥ III (Papile’s classification) [14], cystic periventricular leukomalacia [15], cerebellar haemorrhage, post-haemorrhagic ventricular dilatation, or cerebral atrophy. The exploratory outcomes are (1) major neonatal morbidities associated with neurodevelopmental impairment later in life (count; 0 to 3) [16], (2) bronchopulmonary dysplasia (dichotomous), (3) retinopathy of prematurity stage 3 and above (dichotomous), (4) late-onset sepsis (dichotomous), and (5) necrotising enterocolitis stage 2 and above or focal intestinal perforation (dichotomous).

## General analysis principles

Statistical analyses will be performed using Stata (StataCorp LLC, Texas, USA). Analyses in Stata 18 will follow the recommendation from the Stata Bayesian Reference Manual [17]. All randomised participants will be included in all analyses (intention-to-treat principle), and stratification variables will be included in all analyses (site and gestational age).

### Rationale for Bayesian analyses

The conventional frequentist analyses of randomised clinical trials are often reported using an effect estimate, a confidence interval, and a *p* value*.* Despite advice to the contrary, the results are often interpreted in a dichotomous matter, based on the *p*-value chosen as threshold for ‘statistical significance’ and ‘no evidence of effect’ is confounded with ‘evidence of no effect’.

Bayesian analysis may provide information of the probability of benefits and harms, which may be more easily interpretable [18] and may be less susceptible to the long-standing tradition of misinterpreting results achieved using frequentist statistics [19].

### Priors

The primary Bayesian analyses will use default non-informed priors as defined in Stata—*normalprior*—with a standard deviation of 10, which will be centred around no difference (i.e. centred around 0) and thus including all plausible effects. These default non-informed priors will also be used for covariates. As secondary Bayesian analyses, we will use an informative prior distribution generated from previous randomised clinical trials [20]. The included trials are based on information obtained through a systematic review [21].

### Statistical analyses

The SafeBoosC-III trial has one primary and five exploratory outcomes. The outcomes are dichotomous, apart from one exploratory being a count outcome. The results from the analyses are in Bayesian statistics referred to as ‘posteriors’ and will be presented as point estimates and 95% credible intervals (CrIs), encompassing the most probable results based on the data, model, and priors selected.

#### Dichotomous outcomes

Dichotomous outcomes will be analysed using Bayesian logistic regression models with gestational age and site as control variables (or covariates). Effects on dichotomous outcomes will be presented as the adjusted relative risks (aRRs), adjusted odds ratios (aORs), and adjusted risk differences (aRDs), by comparing the probability of the outcome in the two intervention groups. We will calculate aRRs by applying exponential transformation to the simulated values and then summarise them [17]. Furthermore, we will estimate the probability of any benefit or harm and the probability of clinically important benefit or harm (see the ‘Interpretation of results’ section) (Table 1).

**Table 1:** Presentation of an outcome

	Primary outcome
*Control group*—*% (95% CrI)*	50% (50 to 50%)
*Intervention group*—*% (95% CrI)*	50% (50 to 50%)
*Relative difference*—*aRR/iRR (95% CrI)*	*1.00 (1.00 to 1.00)*
Absolute difference—*RD/MD (95% CrI)*	*0.00 (0.00 to 0.00)*
Probability of any benefit—%	*50%*
Probability of any harm—%	*50%*
Probability of clinically important benefit—%	*25%*
Probability of clinically important harm—%	*25%*
Probability of no clinically important difference—%	*50%*

#### Count data outcome

The count data outcome, major neonatal morbidity count, ranges from 0 to 3 and will most likely present with a non-normal distribution. We will use Bayesian linear regression model with gestational age and site as covariates, corresponding to the planned primary frequentists analyses [7]. The large sample size is most likely sufficient to account for the non-normally distributed data [22]. Effects on the outcome will be presented as a mean difference (MD), by comparing the two intervention groups. Furthermore, the probability of any harm or benefit, together with the clinically important benefit and harm (see the ‘Interpretation of results’ section), will be presented (Table 1).

### Model settings

**Fig. 1:**
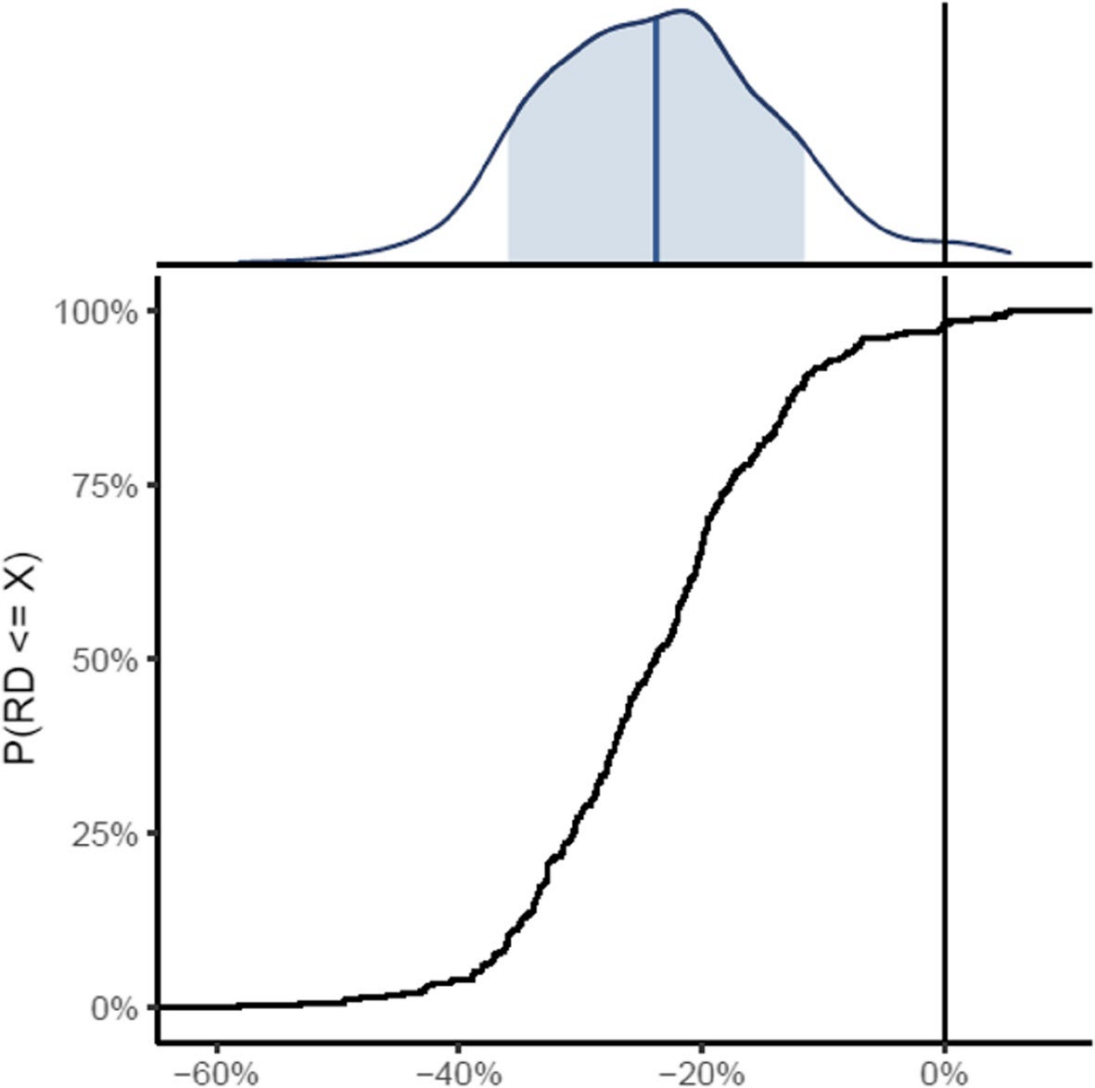
Presentation of a model diagnostic plot including a trace plot, histogram, autocorrelation plot, and density plot. Trace plot (upper left) should depict relative homogenous static noise without any visualisable patterns. The histograms (upper light) must depict a normal distribution. The autocorrelation plot (lower left) indicates the degree of convergence and good convergence and thereby autocorrelation becomes negligible if a pattern of decrease and ends below 0.1. The density plot (lower right) also illustrates convergence and is interpreted by estimating the similarities between the first and second half

Every analysis will be carried out in accordance with the recommendation described in the Stata manual [17]. The models will use a Hamiltonian Monte Carlo sampler with 1 chain, a sampling of 10,000, and at least 25,000 iterations for the burn-in period [17]. Chain convergence will be evaluated by visual inspection of density, autocorrelation, histograms, and trace plots (Fig. 1) [23]. Trace plot should depict relative homogenous static noise without any visualisable patterns; the density plot illustrates convergence and is interpreted by estimating the similarities between the first and second half. Higher serial correlation typically has the effect of requiring more samples to obtain to a stationary distribution. If upon inspection of the autocorrelation plots looks ‘snake-like’ rather than like a hairy caterpillar, this might indicate that more simulations are required. Furthermore, each analysis will be evaluated in three chains using Gelman-Rubin convergence diagnostics and interpreted using 95 R_u_ which needs to be below 1.01 in order to accept the model [24–26].

If convergence issues occur, we will gradually increase the sampling up to 50,000 and the burn-ins gradually up to 100,000. If this does not solve the issues, we will do the analyses by combining sites. Furthermore, if necessary, we will carry out the analysis with and without the problematic covariate(s) and present both analyses [17].

If the initial diagnostic inspection for convergence has proven satisfactory, we plan to assess if changes in the definition of the model result in significant changes in posterior inferences. We therefore plan to compare models with plausible but different priors (including different distributions) and to explore the consequences of inclusion or exclusion of different variables (https://m-clark.github.io/bayesian-basics/diagnostics.html).

### Interpretation of results

The results will be presented as probability of any benefit or any harm, Bayes factor, and the probability of clinical important benefit or harm. Benefit will be defined as the probability that the adjusted RR (aRR) is below 1.0. Harm will be defined as the probability that the aRR is above 1.0. The probability of benefit or harm will be interpreted as high probability, if above 99%. Bayes factor will be estimated for all outcomes, and the Bayes factor described by Jakobsen and colleagues above 10 will be interpreted as a high probability of conformation of the null hypothesis [27]. For the primary outcome, clinically important benefit will be defined as the probability that the aRR is below 0.90, and clinically important harm will be defined as the probability that the aRR is above 1.10 for the primary outcome [7]. Sensitivity analyses using 0.97, 0.95, 0.85, 0.80, 0.75, and 0.70 as benefit and 1.03, 1.05, 1.10, 1.15, 1.20, 1.25, and 1.30 as harm will be carried out. For the exploratory outcomes, major neonatal morbidities associated with neurodevelopmental impairment later in life, bronchopulmonary dysplasia, and late-onset sepsis, an RR of 0.80 and 1.20 will be used to assess clinically important benefit and harm, respectively. For retinopathy of prematurity stage 3 and above secondary, an RR of 0.70 and 1.30 will be used. For necrotising enterocolitis stage 2 and above, an RR of 0.83 and 1.17 will be used. All these estimates relate to the primary sample size calculation and power estimations, and the rationale is described in the primary protocol in detail [7].

### Handling of missing data

Missing data for each variable will be presented in detail, and decision about potential multiple imputation will follow the recommendations of Jakobsen and colleagues [28]. In brief, if missingness is less than 5%, we will carry out complete case analysis and present results from a best–worst and worst-best analyses as sensitivity analyses. Best–worst analysis assumes all missing data in the experimental group has the best possible outcome and those in the control group have the worst possible outcome, and vice versa for the worst-best analysis [28]. Multiple imputation will only be considered if missingness is more than 5%, less than 40%, and missing mechanism is assessed to be missing at random. If relevant, multiple imputation using all stratification variables (i.e. site and gestational age) and selective baseline variables (i.e. birth weight, gestational age, twin (yes/no), and sex) will be carried out. If multiple imputation is carried out, the posteriors will have similar weight in each imputed dataset.

### Statistical reports

The statistical analyses are prepared with predefined coding for Stata (Supplemental material). The report is based on simulated data, which outlines the analyses chosen for the manuscript. Two statisticians will analyse the data independently following this protocol, where ‘A’ and ‘B’ refer to the two intervention groups which is randomly shuffled. The chosen analyses are based on this statistical analysis plan and pre-defined statistical code (Supplemental material). The results from the outcomes will be presented in two independent reports, which will be compared by the coordinating investigator, the two statisticians, and the co-authors. Based on the consensus from this meeting, a final statistical report will be developed, and based on this report, two abstracts will be written by the steering group: one assuming ‘A’ is the experimental group and ‘B’ is the control group and one assuming the opposite. These abstracts will use the results from the blinded final report, and when the blinding is broken, the ‘correct’ abstract will be chosen, and the conclusions in this abstract will not be revised. Furthermore, all three statistical reports will be published as Supplementary material.

## Results

**Fig. 2:**
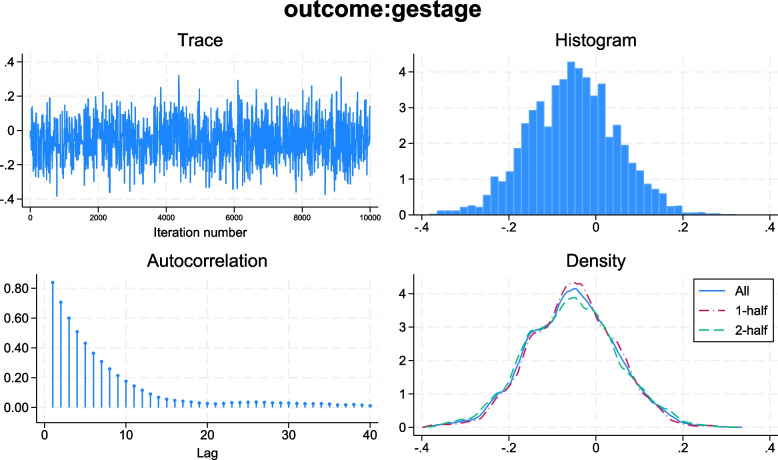
Presentation of an analysis using simulated data. The vertical black line throughout the two plots represents no difference. The upper plot presents the posterior distribution, with the blue area representing the 95% credibility interval and the blue line showing the median value. The lower plot shows the cumulative posterior distribution

See Table 1 and Fig. 2 with simulated data prepared for the final manuscript.

## Discussion

This statistical analysis plan presents a pre-planned secondary Bayesian analysis of the SafeBoosC-III trial. The manuscript will be written after acceptance of our manuscript based on the primary frequentist analysis of the trial [6]; thus, we are able to interpret the findings from two perspectives, both the frequentists and the Bayesian perspective. This pre-planned approach will provide us with the best possible foundation for interpretation of the findings.

The previously addressed, often dichotomous interpretation of *p* values in frequentists statistics does not leave much room for interpretation [19, 29, 30]. Absence of evidence, measured by an insignificant *p* value, is often interpreted as evidence of absence of effect [19, 31]. This absence of evidence could merely be caused by a smaller effect size than the a priori estimated effect size. Thus, the more easily interpretable probability estimates in Bayesian statistics open for the possibility of clinicians and non-statisticians to interpret the results [32], especially the ability to present the probability of different definitions of benefit and harm may help inform future guidelines.

### Strengths

The SafeBoosC-III trial has several strengths, which have previously been described in detail [5, 7]. In brief, the SafeBoosC-III trial is the largest trial in the field [6, 23], has a strict outcome hierarchy [27], and is accompanied by a pre-published design manuscript and frequentist statistical analysis plan [5, 7]. Moreover, the detailed handling of missing data and innovative central data monitoring process further increases the data quality of the trial [33]. The present Bayesian statistical analysis plan describes the analyses in detail, which will minimise the risks of selective reporting bias [34–36]. The details provided above are of a level that independent statisticians should be able to reproduce the statistical analyses [37, 38].

### Limitations

The primary limitation of the SafeBoosC-III trial is the inherent difficulty to blind the intervention. This limitation has previously been addressed [5], but the potential bias has been mitigated by having blinded statisticians and blinded assessment of the subjective component of the primary outcome, severe brain injury, together with the blinded interpretation of results and formulation of the abstracts. The priors introduced by the Bayesian analyses might introduce bias; however, using weakly informed or uninformed priors as primary and evidence-based and sceptic priors as sensitivity analyses aims to minimise the influence of this potential bias.

## Conclusion

This Bayesian statistical analysis plan for the SafeBoosC-III trial includes a detailed predefined description of how data will be analysed and presented for our secondary analyses. We have included detailed descriptions of the statistical considerations aimed to limit selective reporting bias. This statistical analysis plan will likely increase the validity of our results.

### Supplementary Information

**Additional file 1.** 

## Data Availability

The datasets will be made available after reasonable request to the steering committee of SafeBoosC-III, while the scripts for the reports will be made available after reasonable request to the corresponding author.
