# Supplementary material for: Detailed statistical analysis plan for a secondary Bayesian analysis of the SafeBoosC-III trial: a multinational, randomised clinical trial assessing treatment guided by cerebral oximetry monitoring versus usual care in extremely preterm infants
Source: Trials. 2023 Nov 16;24:737. doi: 10.1186/s13063-023-07720-3 (PMC10655478; doi:10.1186/s13063-023-07720-3)
Supplement: Supplementary file 1 — Additional file 1. [file 13063_2023_7720_MOESM1_ESM.docx]

* Import dataframe

import delimited "C:\Oel\Artikler\CTU\CTU_SafeBoosC-III\Bayes-SAP\!old\5918.csv", numericcols(6 7 8 9 10 11 12)

* Ensure dataframe fullfills criteria for analysis

encode site, generate(site_num)

rename var1 arm

* Mixed effect logistic regression

xtmelogit outcome arm gestage || site:, or

* Fixed effect logistic regression

logistic outcome i.arm i.gestage site_num, or

* Calculation of relative risk

margins i.arm,post

nlcom _b[1.arm]/_b[0.arm]

* Bayesian analysis

bayes, normalprior(10): logistic outcome i.arm gestage site_num

* Overall Diagnostics

bayesgraph diagnostics _all

bayesstats ess

* Calculation of Gelman-Rubin statistics for diagnostics

net install grubin, from(http://www.stata.com/users/nbalov)

bayesmh outcome i.arm gestage site_num, prior({outcome:}, normal(0,10)) mcmcsize(20000) saving(model1,replace) initial({outcome:} 0) likelihood(logit)

estimates store M1

bayesmh outcome i.arm gestage site_num, prior({outcome:}, normal(0,10)) mcmcsize(20000) saving(model2,replace) initial({outcome:} 10) likelihood(logit)

estimates store M2

bayesmh outcome i.arm gestage site_num, prior({outcome:}, normal(0,10)) mcmcsize(20000) saving(model3,replace) initial({outcome:} -10) likelihood(logit)

estimates store M3

grubin, estnames(M1 M2 M3)

* Clinical benefit, harm, and futility

bayestest interval (AnyHarm:exp({outcome:1.arm}), l(1)) (AnyBenefit:exp({outcome:1.arm}), u(1)) (ClinBen:exp({outcome:1.arm}), u(.95)) (ClinHarm:exp({outcome:1.arm}), l(1.05)) (Futility:exp({outcome:1.arm}), l(.95) u(1.05))

* Calculation of Bayes factor

bayes, normalprior(10) saving(model1,replace): logistic outcome i.arm gestage site_num

estimates store M1

bayes, normalprior(10) saving(model2,replace): logistic outcome gestage site_num

estimates store M2

bayesstats ic M2 M1, bayesfactor
